# How does it end? Endpoints of boundaries lead to completion in macro-events

**DOI:** 10.3758/s13421-024-01657-x

**Published:** 2024-11-15

**Authors:** Ayşe Candan Şimşek, Tolgahan Aydın, Markus Huff

**Affiliations:** 1 Leibniz-Institut für Wissensmedien, Tübingen, Germany; 2 Eberhard-Karls-Universität, Tübingen, Germany

**Keywords:** Event completion, Predictive processing, Backwards inferences, Event boundaries, Macro events

## Abstract

While watching someone kicking a ball, missing moments of ball contact can be incorrectly identified as seen if the event is continued in a causal manner (i.e., the ball flying off). Does event completion also occur for events of a larger scale such as having breakfast (macro-event), which consists of multiple sub-steps like toasting bread (micro-event)? We conducted two experiments to measure event completion in macro-events presenting portions of multiple micro-events. In Experiment 1, video summaries were formed with or without event boundary information where a macro-end was either present or absent. Macro-end signified an overarching goal achievement that signaled the completion of previous tasks (such as leaving the kitchen with a full breakfast plate). More completion occurred for summaries with event boundary information and macro-ends. In Experiment 2, we tested two alternative hypotheses to explore the underlying process by showing the beginnings or ends of a micro-event. While the predictive processing hypothesis suggests that event completion is based more on predicting the future states of the event based on beginning information, the backward inferences hypothesis suggests that event completion relies more on deductions formed after the fact based on event endings. Results of Experiment 2 suggest that the ends of event boundaries lead to more event completion, possibly due to their role in forming causal connectivity. These results help to further understand event completion on a macro level.

**Supplementary Information:**

The online version contains supplementary material available at 10.3758/s13421-024-01657-x.

## Introduction

Imagine watching the movie *The Matrix* (Wachowskis, 1999) at home on a Sunday night. Neo is at the train station with Agent Smith, and they are flexing their fingers, getting ready for action. Your phone rings and you answer the call. After a while, you get back to the movie and you see Agent Smith getting crushed under a train. Neo must have won this fight. Event completion studies suggest that you tend to fill in the missing information if the narrative is coherent. When you see the fight scene later, you might think that you already saw it, even though you didn’t. But the question must be asked: Would this process depend on whether you saw Agent Smith getting crushed under a train or not? Or would this process depend on the structure of actions you observed while you watched the film? In this study, we try to answer what leads to event completion in complex daily events.

*Event completion* is a phenomenon where people form coherent mental models in the face of missing information. It suggests that people are prone to filling in the blanks of missing information when the visual input is causally continuous (Brockhoff et al., 2016; Papenmeier et al., 2019; Strickland & Keil, 2011). In their original work, Strickland and Keil (2011) presented participants with naturalistic events where the final action was either causally or non-causally connected. The authors used short clips where a football player places a ball on the grass, runs, and swings out his leg, and the videos either included the contact moment or that this segment was missing. People produced *more false alarms* to the contact moment when there was a causal continuation (i.e., the ball flying). This tendency leads to confusion concerning action components that fit well within a continuous framework and suggests that people form more false alarms for segments that fit better with a coherent stream of events in a way similar to amodal completion (Kanizsa, 1979: Kaup et al., 2022). This suggests that when there is causal continuation, people adjust their event model accordingly to include the missing information. Building on earlier work, Brockhoff et al. (2016) also presented soccer clips to participants with the ball contact moment either present or absent. They used novices as well as experts to examine top-down influences on event completion. When the missing information was followed by a causal completion, people falsely reported seeing the missing segment. As this effect was present for both novices and experts, we can argue for an experience-free completion of event models in response to missing information. Similarly, novel events were also shown to create false memory, arguing for event completion for familiar as well as unfamiliar events (Komisky et al., 2021).

Researchers also questioned how time estimation was affected when an event is incomplete. In a related work, Garsoffky et al. (2017) investigated whether the temporal nature of the missing information affects judgments of event duration. The researchers used a series of sequences involving four shots, which had either short or long gaps. Videos with both short and long gaps ended with the same target shot, but the temporal distance between slices of events involved either short or long gaps. People’s estimation of how long the target shot would take in daily life was longer in sequences involving long gaps. Studies also indicated that people form higher-level representations of events when they are exposed to long gaps within the event (Garsoffky & Schwan, 2020). The length of temporal gaps also affected people’s predictions of future event segments. When asked to *extrapolate* to predict the upcoming action, people again used higher-order descriptions when the activity contained long gaps. When exposed to incomplete information, people form coherent representations that include a differing level of detail. Importantly, however, those studies did not test for detection but used event comprehension measures. These findings suggest that observers form a more extensive continuous representation that includes the segments they have not experienced but still fit with the coherent event.

As event cognition operates on predictions and backwards inferences, which one applies more to event completion is not fully understood. To examine this question, Papenmeier et al. (2019) asked questions about a missing event segment immediately after the presentation. The authors showed that false memory was present after an event is experienced, which suggests that event completion happens very quickly and may be based more on *backward inference* and less on *predictive processing*. Literature provides evidence for backwards inferences mostly in written narratives (Graesser et al., 1994; Haviland & Clark, 1974, Kintsch, 1988, 1998; Singer & Ferreira, 1983). Accordingly, people complete the missing information in a top-down manner based on previous knowledge about the world. Contrary to predictive processing, backwards inferences fill in the missing information based on what comes next, not on what comes before. Schmalhofer et al. (2002) proposed a “Unified Model for Predictive and Bridging Inferences,” which gives the role of backwards inferences a theoretical context. In this model, various word possibilities are activated in the construction phase in a top-down manner, and which of those will be eliminated is based on the subsequent information in the integration phase. Singer (1996) also suggested a theory focusing on backwards inferences in discourse processing. Backwards inference in this theory works as an experience-based deduction that is formed after the fact from given information. Research on bridging inferences shows that people need to put effort into inferring missing information, affecting working memory resources (Fincher-Kiefer & D’Agostino, 2004; Magliano et al., 2017). This process not only applies to discourse processes but also to visual narratives. For example, using picture stories, Magliano et al. (2016) found evidence in favor of backwards inferences, showing that viewing times were longer for an end-state event if the bridging event was absent.

The definition of an event is still a matter of debate. The notion of what an event is and what it includes is not yet resolved in the literature (Ji & Papafragou, 2020, 2022; Yates et al., 2023). One of the most referred-to definitions of an event is “a segment of time at a given location that is perceived to have a beginning and an end” (Zacks, 2004). Although events are conceptualized as discrete units, they are also qualified as infinitely divisible and embedded (Yates et al., 2023). Previous research camped around two influential theories concerning how people perceive event boundaries in ongoing events. Based on primarily written narratives, the *Event Indexing Model* (Zwaan et al., 1995) offers one of the earliest characterizations of event cognition. Mental representations obtained through *event models* are formed to process information according to already existing schemas. People monitor events along multiple dimensions (time, space, actor, goals, and causality), and when a considerable amount of change is presented along one or more dimensions, a new situation model is created. Some research argued in favor of the additivity hypothesis, indicating that if more change happens along multiple dimensions, people’s memory for content was better, but in contrast, their predictions were less correct for what came next (Huff et al., 2014).

Building off the key elements of the *Event Indexing Model*, *Event Segmentation Theory* (EST) (Radvansky & Zacks, 2011: Zacks et al., 2007; Zacks et al., 2009) adapted the event model idea to everyday events and visual narratives in general as an *experience model*. Event models can be divided into situation models (Zwaan, 2016) and experience models (Radvansky & Zacks, 2011). As situation models are characterized as models based on written narratives, experience models refer to lived experiences. EST is a computational model stating that people form *dynamic* event models in working memory that are continually updated based on the incoming information. People make predictions about the next steps in an event until there is an event boundary that requires the activation of a new event model (Zacks, 2020). Event models serve as a source of *predictions* for upcoming information. If the information is consistent with predictions, they are incorporated into the current schema and, if not, a break occurs in the perceptual continuity and an event boundary is perceived (Zacks et al., 2010). Research so far has shown that event boundaries contain essential information about an activity (Michelmann et al., 2023). As there is considerable agreement on event boundaries, these are also associated with higher attention and recall (Huff et al., 2012; Swallow et al., 2009). One reason for this effect was attributed to increased attention leading to better encoding of information into long-term memory. Note that this increased attention process occurs at the expense of peripheral details (Huff et al., 2012).

Further theories questioned the nature of the predictions and whether each change in an event model creates a prediction error (Shin & DuBrow, 2021). Accordingly, such a theory suggests that event segmentation is an inference process, where each upcoming event has a different likelihood to follow a previous event. For example, washing clothes is more likely to be followed by hanging clothes on a drying rack than putting a record on a turntable. So, even though inference accounts are not incompatible with theories like EST, each prediction error is considered to have different intensity based on causal structure changes.

A recent account of the concept of event boundedness can help distinguish the importance of event boundaries and non-event boundaries for the event completion. Bounded events such as “building a sandcastle” involve a non-homogeneous structure that allows distinct sub-actions to be defined, while unbounded events such as “playing with sand” involve a homogeneous structure that makes it hard to separate temporal slices from one another (Ji & Papafragou, 2020, 2022). Such a conceptual difference privileges event endings (event boundaries) more than event midpoints (non-event boundaries) when events are bounded (Ji & Papafragou, 2020). It also makes it difficult for the viewer to notice disruptions during event endings, compared to event midpoints only for bounded events that have internal structures (Ji & Papafragou, 2022). However, current literature cannot explain whether event endings are also privileged with bounded events that lack overarching natural ending, or whether event beginnings of bounded events are different to event endings.

Previous research on event cognition consistently used the methodology of asking people to segment a stream of information into *fine* and *coarse* events, which are deemed to reflect a hierarchical structure (Magliano & Zacks, 2011; Radvansky & Zacks, 2014; Zacks et al., 2007). As fine events can be considered more perceptual, coarse events can be considered more conceptual (Zacks et al., 2009). Also, some accounts stress that events are infinitely divisible (Yates et al., 2023), so identifying coarse and fine boundaries can be seen as a relative process. Consider the routine of preparing breakfast in the morning. Each activity (i.e., preparing tea, buttering bread, having oatmeal) can be considered as a coarse event, whereas each sub-activity (i.e., taking a mug from a cupboard, boiling water, etc.) can be considered as a fine event. We suggest that event granularity is relative and should be considered accordingly. Take the breakfast preparation example again. If we consider this activity as part of a morning routine, which includes other activities (i.e., getting up, taking a shower, dressing, etc.), then preparing breakfast itself becomes a coarse event and preparing tea a fine event. Accordingly, we would like to propose two concepts regarding the structure of events. The concept that we will call a “macro” event is a combination of what we will call “micro” events. As such, a macro-event encloses multiple micro-events. Similar to the partonomic structure of granularity (Garsoffky & Schwan, 2020), micro-events emerge as parts of a whole, namely macro-events. Here, we consider micro and macro-events to fall under a spectrum where macro-events are coarser compared to micro-events, which are finer. In that regard, we can consider preparing breakfast or cleaning a room as examples of macro-events, while dusting a shelf or buttering toast as examples of micro-events. We should make it clear though that a micro-event, while finer, can still be divisible into further segments. As these terminologies are still new and need further exploration, further research is still needed to disentangle their nature.

**Fig. 1:**
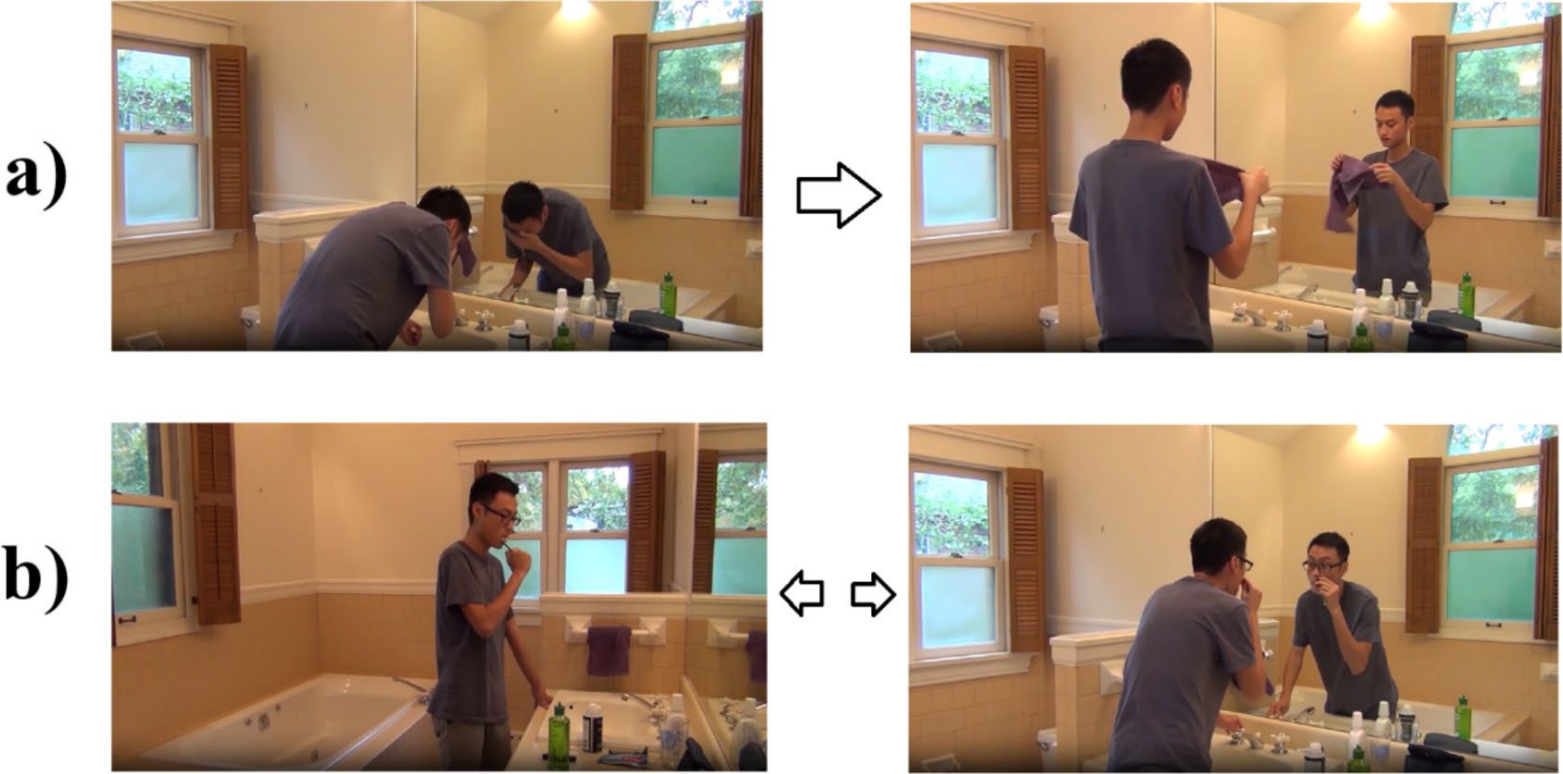
A comparison of micro and macro-events in terms of order. While micro-events like (**a**) washing someone’s face must have the necessary order of washing one’s face before drying it, order of a macro-event like (**b**) bathroom routine can include interchangeable segments like brushing one’s teeth and shaving

The order of events is another dimension that helps us define macro- and micro-events. We suggest that the order should be considered on a continuum. Referring to the stimulus material of Bezdek et al. (2023), we consider macro-events to be less order-bound (i.e., you can prepare tea before or after preparing oatmeal), while micro-events to be more order-bound (you should toast the bread before spreading cream cheese) (see Fig. 1). One reason for this can again be the nature of coarser events to be more conceptual, while finer events to be more perceptual.

One other difference between micro and macro-events can be the existence of a causal ending. For the macro-event *bathroom routine*, multiple end scenarios are possible. For example, it can end with shaving or brushing teeth. Thus, a macro end is more bound by the completion of multiple goals that can imply the ending of multiple micro-events. On the other hand, micro-events in our stimuli have a more specific (causal) ending. For example, the micro-event of shaving is bound to washing one’s face at the end. In addition, for a naïve viewer who does not know what constitutes the bathroom routine of the actor, the ending of each micro-event can also signify the beginning of another micro-event unless there is other information that signals the end of the macro-event category. For example, the person leaving the bathroom. To signal such a macro end, we planned to add one last scene where the actor leaves the room.

Finally, event completion is an essential phenomenon in human cognition as it allows human observers to make sense of events that cannot be continuously attended to. This may help people to develop an understanding of the world, and to remember the details of an event by providing a sense of continuity despite the missing input. It is still an open question which factors are of interest when people complete macro-events. Of interest to this project, we inquired how event boundaries – the transitions between two meaningful units – and different event portions (beginnings and ends) affect event completion. We present the first evidence that event boundaries and more specifically the end portion of events impact completion of macro-events.

### Experimental overview and research questions

We report two experiments investigating event completion in macro-events and the importance of event boundaries. Participants watched event summaries and performed a detection test afterward. The objective of Experiment 1 was to examine the effect of macro end (present vs. absent) and event boundary information (with vs. without) on the detection memory of macro-events. Research showed that event completion increases when a micro-level event is causally continuous (Strickland & Keil, 2011). While a macro end may not be a necessary causal ending, it still signals completion of multiple goals. In that regard, we expect that the presence of a macro end would also trigger more event completion compared to its absence. Also, with respect to the relationship of event boundaries to event completion, Schwan and Garsoffky (2004) showed that it is sufficient to watch *event summaries* containing only the event boundary information for understanding macro-level activities. This demonstrated that event summaries, including boundary information, were perceived as similar to complete events. Accordingly, we hypothesized more event completion (i.e., lower sensitivity and higher false alarms to not-seen parts of the event) if the event summaries depicted a macro end and an event boundary (vs. non-event boundary) information.

In Experiment 2, we tested the underlying process of the event completion effect observed in Experiment 1 by comparing beginnings and ends of micro-events across event boundaries and non-event boundaries. On the one hand, previous research favored the beginning of events to be more crucial to the mental models. In such a study, Teigen et al. (2017) showed what they call a “beginning advantage” where people were exposed to epochs and events in an historical timeline as well as cyclical change in annual seasons. The beginnings of historical events (i.e., wars) were judged to be more important and people were more engaged and interested in reading about them. Researchers interpreted the findings as such that the beginning of events include more changes, attract more attention and contribute more to causality. This suggests that beginnings might have more informational value. However, not being able to identify the end of an event might prevent updating mental models, leading to an inadequate event model. In our stimuli, each sub-segment, which can also be referred to as a micro-event, had a beginning and an end. Whereas the beginnings of micro and macro-events are hard to distinguish (e.g., getting a cup to make coffee could be the beginning of a micro-event “getting the cup” or a macro-event “preparing breakfast”), the end of micro and macro-events are clearly distinguishable (i.e., by knowing its history). Accordingly, our hypotheses were twofold. If event completion is driven more by predictive processing (Radvansky & Zacks, 2017; Zacks, 2020) – humans fill in the missing parts by predicting the future states of the event – the *beginning* of an event should trigger more event completion. However, if it is fast based more on backward inferences – humans fill in the missing parts based on fast backward inferences – the *end* of an event should trigger more event completion (Papenmeier et al., 2019).

## Experiment 1

Experiment 1 investigated the role of event boundaries and macro ends on how well people remember details of macro-level activities (i.e., cleaning a room). Event boundaries were presented as within-subjects variables: each video either included event boundaries or non-event boundaries. Macro end (present vs. absent) was presented as a between-subjects variable. We expected more false alarms to missing segments (hence the lower sensitivity d’) when event boundaries or macro ends are present. These results imply more event completion, which signifies more coherent models of event representations.

The pre-registration for Experiment 1 is available at https://aspredicted.org/9c7mb.pdf.

### Method

#### Participants

Participants were recruited at Yaşar University and Eberhard Karls University of Tübingen. A power analysis using G*Power (Faul et al., 2007) with a power of .80, alpha level probability of .01, effect size f .15 gave the minimum number of 82 participants. We collected data from a total of *N* = 137 participants. In summary, 110 participants from Yaşar University (93 female, 17 male; 19–38 years old, *M*_*age*_ = 22.9 years) and 27 participants from Eberhard Karls University of Tübingen (17 female, nine male, and one non-binary; 19–32 years old, *M*_*age*_ = 21.6 years) took part in the experiment in exchange for course credit. We did not expect any differences between the two samples since we did not expect the studied basic processes of event cognition to depend on culture. Additionally, the stimuli used in two studies did not involve culturally irrelevant action sequences and they were everyday events (such as breakfast or checking one’s phone) that can be considered normal for both Germany and Turkey. Participants performed the experiments in their native language.

We pre-registered data exclusion based on the boxplot criterion for the dependent variable d’ on the participant level. Based on this criterion, no participant had to be excluded from the data analysis.

#### Materials

We used eight different videos from the Multi-angle Extended Three-dimensional Activities (META) stimulus set by Bezdek et al. (2023) to create our stimuli. This stimulus set is publicly shared to study event segmentation of naturalistic activity. We executed segmentation magnitude analysis for eight videos using the SegMag procedure (Papenmeier & Sering, 2016) with the data of Bezdek et al. (2023) to define places of event boundaries. The segmag package uses a bootstrapping approach to identify significant event boundaries as those points in time, where the agreement across participants’ segmentation responses is above the critical threshold that could be expected by chance. Segmentation magnitude analysis was implemented by setting the critical threshold to 99% and with 1,000 iterations. Hence, the places of event boundaries corresponded to seconds (bins) in the video which most participants agreed. There were, on average, 6.13 coarse event boundaries per video. We selected the coarse boundaries because they relate to macro-level events. Places of non-event boundaries were defined as the middle of two event boundaries. Following that, we created event summaries with event boundaries and non-event boundaries.

Event summaries formed from event boundary information included shots only taken from event boundaries (4 s before and 4 s after a coarse boundary). Event summaries formed from non-event boundary information included shots only taken from the middle point of a coarse segment (4 s before and 4 s after the midpoint of a coarse segment). It was ensured that each shot included only one coarse segment and that boundaries did not overlap. The event summaries were created using alternating shots from two different angles to avoid jump cuts, and smooth transitions in the form of fade-in and fade-out were used instead of straight cuts. The event boundaries used here are based on event segmentation data from another sample. This methodology has been implemented by various previous literature (Schwan & Garsoffky, 2004; Schwan et al., 1998), where a pre-segmentation of the videos is used as source for event boundaries.

**Fig. 2:**
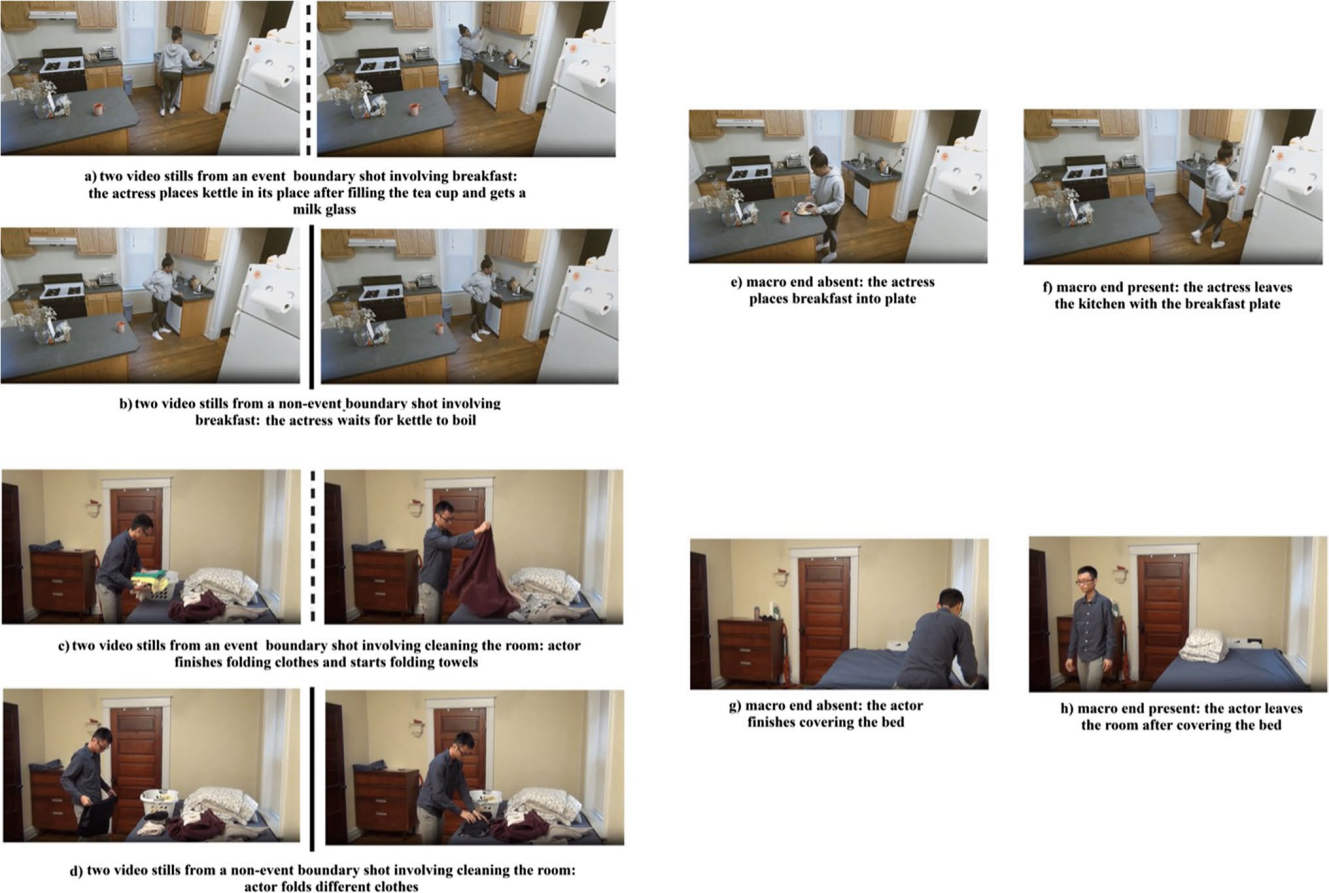
Video stills from event boundary and non-event boundary shots with and without macro ends. Example videos of all conditions of Experiment 1 can be found at https://www.youtube.com/playlist?list=PLEpYiT6MiZy7Au5ZDerKzcLBYL1ZUSJha. Video stills (**a**) and (**c**) show two scenes from event boundary shots while video stills (**b**) and (**d**) show two scenes from non-event boundary shots. Additionally, video stills (**e**) and (**g**) show conditions without macro ends while video stills (**f**) and (**h**) show conditions with macro ends. The dashed and solid lines show the centers of event and non-event boundary shots obtained through the segmentation magnitude analysis, respectively. Shots with event boundaries involve larger changes in actions (folding clothes and folding towels) than shots with non-event boundaries (folding different clothes). The presence and absence of macro ends are reflected on the right-hand side of the figure. Where a macro end is absent, the event only includes the ending of the last micro-event (covering the bed), where a macro end is present however, the event also includes the end of the overarching macro-event (leaving the room)

In addition, half of the stimuli depicted a macro end (control condition) while the other half lacked a macro end (treatment). In Strickland and Keil (2011), a causal continuation included the end of the action that included the immediate effect of the contact moment. Since we investigated event completion with macro-events with higher order organization of multiple sub-events, we manipulated the presence of the ending segment in each complete video. More specifically, event summaries with macro ends included the last 8 s of the original META videos (e.g., for the breakfast video, the actor exits the kitchen carrying out the prepared items). Event summaries without macro ends did not include the last shot showing the ending of the activity. The duration of the event summaries was 69 s on average. (*SD* = 12.34; range: 48–102 s). Figure 2 below shows the differences in stimulus types.

Target and distractor items (i.e., signal and noise) were 4 s long. Target items depicted the middle of each shot of the seen event summary clip. Distractor items for event boundary clips depicted the middle of the corresponding non-event boundary summary. Distractor items for non-event boundary clips depicted the middle of the corresponding event boundary summary. We tested visual detection performance for each clip with *N* = 117 items (59 targets, 58 distractors). Unequal numbers of event and non-event boundaries caused the difference in the numbers of test and distractor items. This was due to our method of determining the non-event boundaries. Shots without event boundaries outnumber shots with event boundaries by one. However, the chosen counterbalancing procedure, which ensures that all videos were presented in each condition, controlled for this difference. Each activity type was presented in all formats (in event boundary and non-event boundary summaries where the macro end is either present or absent) across different groups.

#### Procedure

The experiment was executed as an online experiment using the Pavlovia platform of PsychoPy (Peirce et al., 2019). After providing informed consent and demographic information, participants received instructions about the experimental procedure. They then continued to a practice session with two trials. The main experiment consisted of eight trials. A trial started with participants watching an event summary and a subsequent detection performance test. In the latter, participants had to decide whether clips from each shot (4 s coming from the middle) was part of the video summary or not by pressing the arrow keys (*right* key for “yes” and *left* key for “no”). In the practice session, participants received feedback for their responses. Following the last video of the experimental session, they were thanked, and the experiment ended.

#### Design, independent variables, and dependent variables

The experiment was realized as a 2 × 2 mixed design, including the factors *macro end (present vs. absent)* and *event boundary* (with, without; within-subjects). We counterbalanced the assignment of the event boundary and macro end, so that each activity appeared in each of the four experimental conditions. This resulted in four counterbalancing conditions, of which two were collected in Turkey and the other two in Germany.

We report *false alarms* (proportion of falsely as targets identified distractors items) as the primary dependent variable. In addition, we report *hits* (proportion of correct answers to target items), sensitivity *d’* from signal detection theory (Green & Swets, 1966), and criterion *c* as response bias.

### Results

We fitted linear mixed-effect models with *macro end* and *event boundary* as fixed effects (main effects and interaction) and *participant* as random intercept, separately for each dependent measure using the *lme4* package (Bates et al. 2015). We analyzed the models’ parameters with type-II ANOVAs, using the Anova() function of the *car* package (Fox & Weisberg, 2011). As pre-registered, we considered any value lower than alpha level .05 to be significant.

#### False alarms

**Fig. 3:**
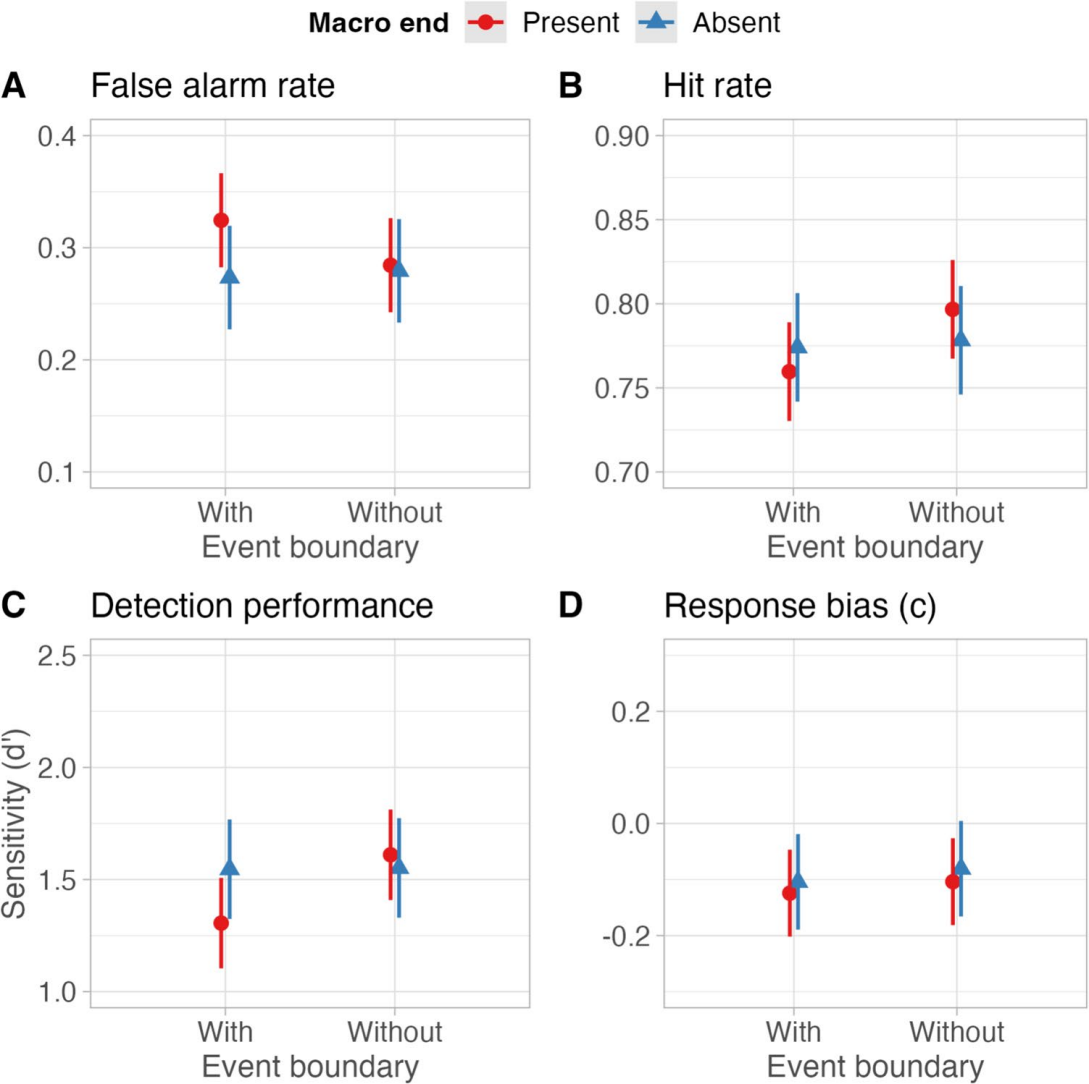
Marginal effects of the interaction term. Error bars represent the 95% confidence intervals

Contrary to our hypotheses, the main effects of *macro end*, χ^2^ (1) = 0.89, *p* = .344, and *event boundary*, χ^2^ (1) = 3.02, *p* = .082, were not significant. However, and most importantly, the interaction of *event boundary* and *macro end* was significant, χ^2^ (1) = 4.29, *p* = .038, with the highest false alarm rate in summaries with event boundary information and macro end. More specifically, for summaries with event boundary information, macro ends led to higher false alarm rates, but for summaries without boundary information, no difference was found for macro ends (see Fig. 3A). Thus, the hypothesized effect was only found when the event summaries depicted both macro end and event boundary information.

#### Hits

The main effect of the event boundary was significant, χ^2^ (1) = 6.15, *p* = .013, with a lower hit rate in the condition with boundaries than in the condition without boundaries. Both the main effect for macro end, χ^2^ (1) = 0.01, *p* = .922, and the interaction of both factors, χ^2^ (1) = 3.34, *p* = .068, were not significant (see Fig. 3B).

#### Sensitivity (d’)

The main effect for *macro end* was not significant, χ^2^ (1) = 0.41, *p* = .524. Yet, *d’* was lower in the condition with event boundaries than without event boundaries, χ^2^ (1) = 10.33, *p* = .001. Most importantly, the interaction of *event boundary* and *macro end* was significant, χ^2^ (1) = 7.98, *p* = .004. As can be seen in Fig. 3C, sensitivity was lowest in event boundary summaries with macro ends, thus reflecting the false alarm rates.

#### Response bias

Neither the main effects of macro end, χ^2^ (1) = 0.18, *p* = .676, nor event boundary, χ^2^ (1) = 0.64, *p* = .423, nor the interaction of both factors, χ^2^ (1) = 0.00, *p* = .955, were significant (see Fig. 3D). Please see Appendix A (Online Supplemental Material) for a detailed view of the estimates for each dependent variable.

### Discussion

We hypothesized more event completion (i.e., a higher false alarm rate) if event summaries had macro ends *or* event boundary information. However, we found more event completion if the event summaries had macro end *and* event boundary information (i.e., the interaction of both factors). The sensitivity (d’) analysis further supports this finding. Thus, event completion for macro-events is more likely to occur if a completed goal involving multiple micro-events *and* event boundary information is available. This result provides a conditional role of event ends in event completion. As causal continuation was linked to more event completion for micro-level events (Brockhoff et al., 2016; Strickland & Keil, 2011), when a macro-event ends, this by itself does not send a large enough signal for people to complete the event series. While a macro end is linked to an overarching goal completion, the event summary still requires event boundary information for a coherent mental model. As event boundaries are consistently linked to better memory in the literature (Zacks et al., 2007; Swallow et al., 2009), they appear to have higher informational value. This can, in turn, be linked to how people use this information to fill in the missing pieces, leading to higher false memory. As boundaries are situated at the end of a previous event segment and at the beginning of the subsequent event segment, Experiment 2 tests which portion is driving the effect.

## Experiment 2

Based on results from Experiment 1, Experiment 2 tested which portion of an event boundary triggers more event completion – the end of the old event or the beginning of the new event. If end portions lead to more event completion, this would suggest that backward inferences affect the formation of event models more (Papenmeier et al., 2019). On the other hand, if the beginning portions of events lead to more event completion, this would suggest that the formation of event models is based more on predictive processing (Radvansky & Zacks, 2017).

The pre-registration for Experiment 2 is available at https://aspredicted.org/nc5nm.pdf

### Method

#### Participants

Participants were recruited at Eberhard Karls University of Tübingen. A power analysis using G*Power (Faul et al., 2007) with a power of .80, alpha level probability of .01, effect size f = .15 gave the minimum number of 88 participants. We collected data from 92 participants (24 male, 93 female; 18–30 years old, *M*_*age*_* =* 20.5 years). First, we excluded two participants with incomplete data. Second, as in Experiment 1, we pre-registered data exclusion to be based on the boxplot criterion for the dependent variable d’ on the participant level. Based on this criterion, two further participants had to be excluded from the data analysis. Thus, the final sample consisted of 88 participants.

#### Materials

**Fig. 4:**
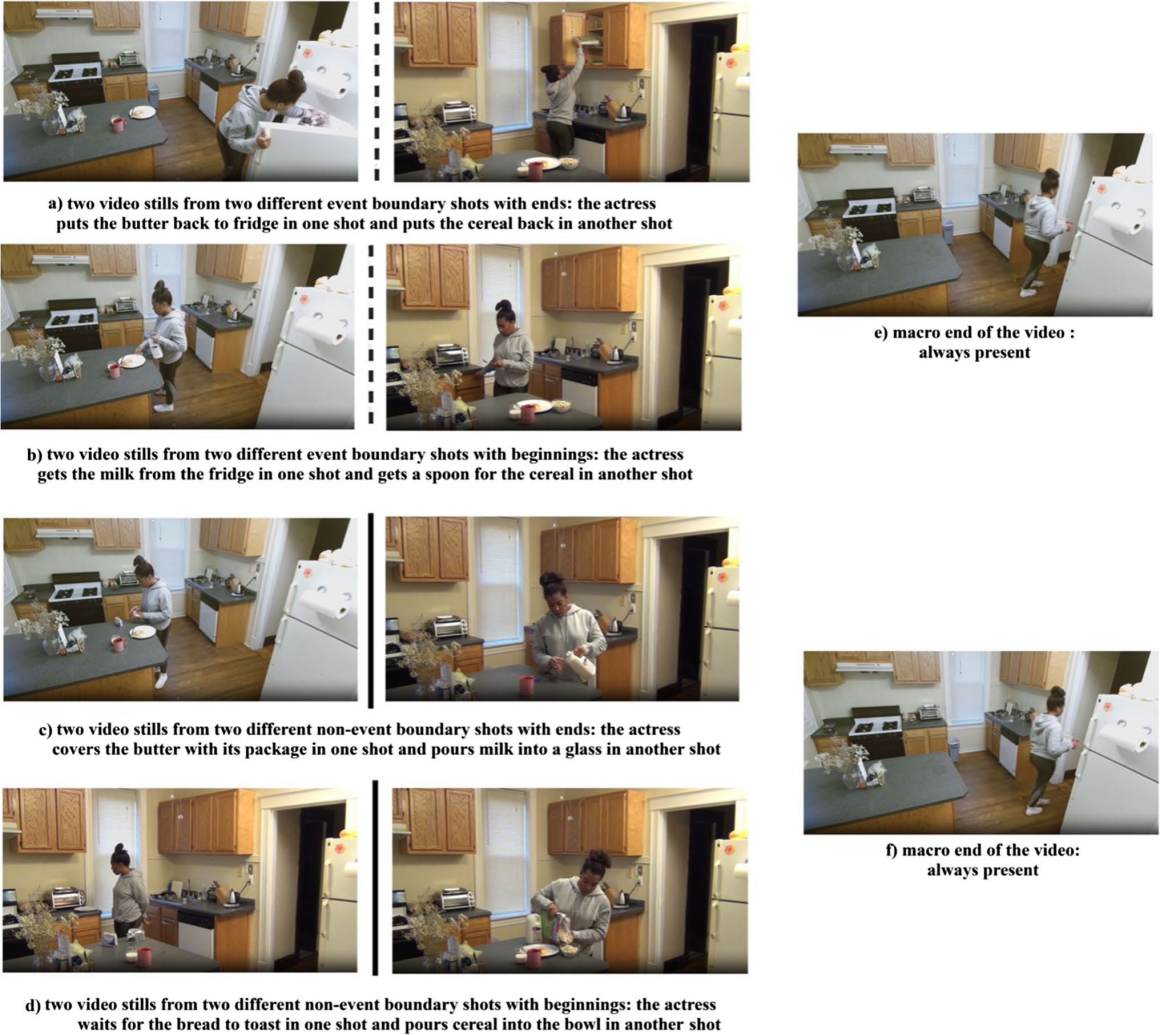
Video stills from event boundary and non-event boundary shots with and without event boundaries. Example videos of all conditions can be found at: https://www.youtube.com/playlist?list=PLEpYiT6MiZy7WpBNXIwAgQ6-n0E0R5KYb. Ends of event boundaries (**a**) can be easily separated from the beginning of event boundaries (**b**), since perception of a new event occurs at event boundaries. On the contrary, ends of non-event boundaries (**c**) do not signify perception of a new event and make it difficult to compare with beginnings of non-event boundaries (**d**). It must be also noted that stimuli in Experiment 2 always involved macro ends (**e**,** f**). The dashed and solid lines show the centers of event and non-event boundary shots obtained through the segmentation magnitude analysis, respectively

The same eight videos from the META stimulus set (Bezdek et al., 2023) were used to create event summaries. Each event summary started with the first 8 s of the video and ended with the last 8 s of the video. The rest of the video consisted of either event boundaries or non-event boundaries. Different from Experiment 1, segments in the video either included the beginning or the end portions of micro-events (see Fig. 4). In the event boundary condition, *beginning portions* were taken from the 8-s segment *after a* coarse event boundary, and the *end portions* were taken from the 8-s segment *before* a coarse boundary. In the non-event boundary condition, the beginning portions were taken from the beginning of the middle point of a coarse segment (i.e., the 8-s segment *before* the middle point of a coarse segment), the *end portions* were taken from the end of the middle point of the respective coarse segment (i.e., the 8-s segment *before* the middle point of a coarse segment). The criteria for creating the event summaries, the target, and the distractor items were the same as in Experiment 1. Participants in each group were tested with 113 items (57 targets, 56 distractors).

The duration of the event summaries was *M* = 74 s (*SD* = 11.25; range: 56–102 s) which is longer than in Experiment 1 because all stimuli in Experiment 2 included the macro end.

#### Procedure

The procedure was similar to Experiment 1 with the following exception. We additionally asked participants to rate how confident they are in their responses, using a 6-point scale (50% – “I was guessing”, 60%, 70%, 80%, 90%, 100% – “I am completely sure”). We did not further analyze this measure in this project.

#### Design, independent variables, and dependent variables

The experiment was realized as a 2 × 2 within-subjects design, including the factors *portion* (beginning, end) and *event boundary* (with, without). We counterbalanced the assignment to the *event information* and *portion* conditions to eliminate any potential influence from the video clips, which resulted in four counterbalancing conditions. Participants were randomly assigned to counterbalancing conditions. We report the same dependent variables as in Experiment 1.

### Results

We fitted linear mixed-effect models with *portion* and *event boundary* as fixed effects (main effects and interaction) and *participant* as random intercept, separately for each dependent measure using the *lme4* package (Bates et al. 2015). We analyzed the models’ parameters with type-II ANOVAs, using the Anova() function of the *car* package (Fox & Weisberg, 2011). As pre-registered, we considered any value lower than alpha level .05 to be significant.

#### False alarms

**Fig. 5:**
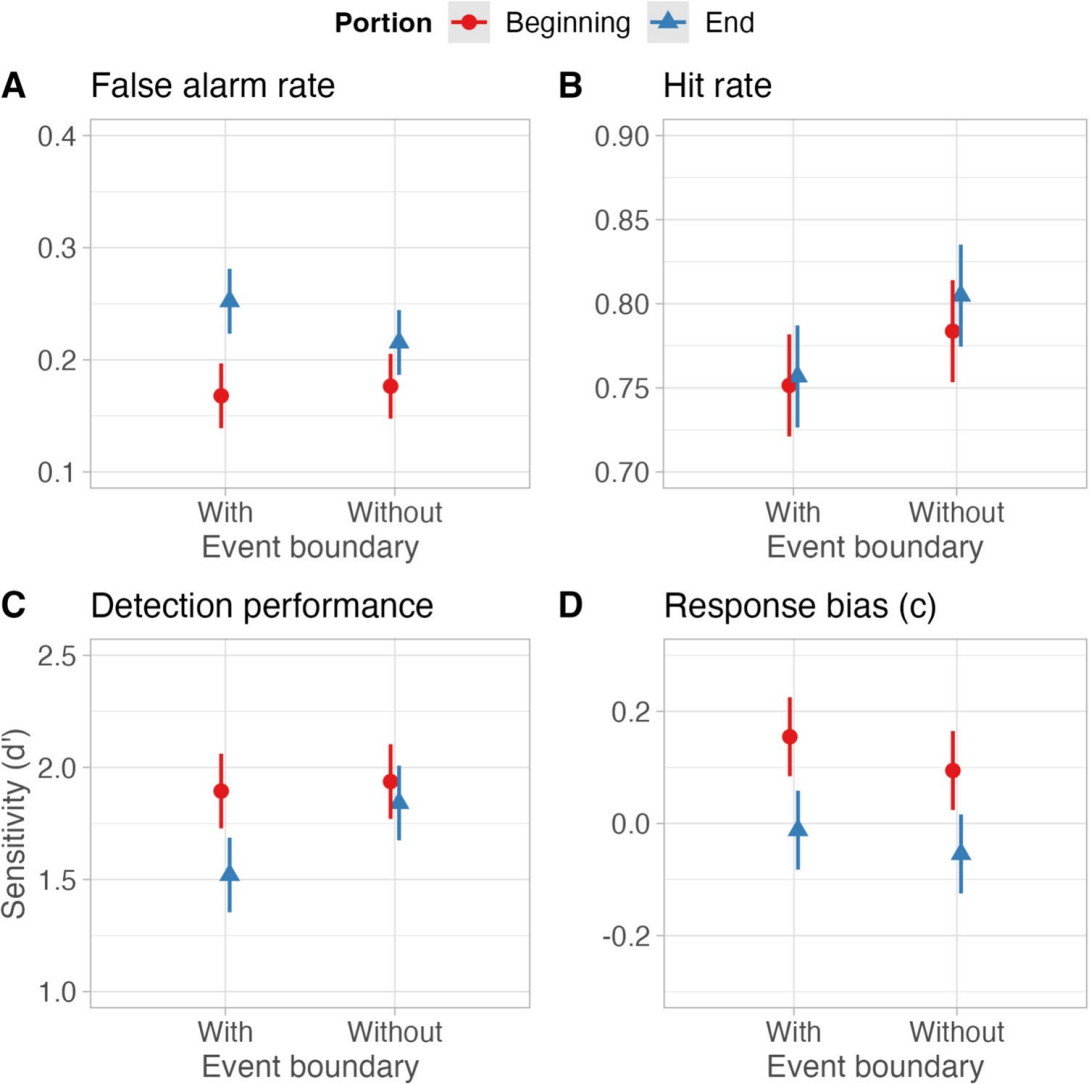
Marginal effects of the interaction term. Error bars represent the 95% confidence intervals

As predicted, the interaction of *portion* and *event boundary* was significant, χ^2^ (1) = 4.89, *p* = .027, with the highest false alarm rate in the condition depicting the end portion of event boundaries (see Fig. 5A). Further, the main effect of event boundary was not significant, χ^2^ (1) = 1.90, *p* = .168, and the main effect of portion, was significant, χ^2^ (1) = 36.25, *p* < .001, with higher false alarm rates in the end portion condition.

#### Hits

The main effect of *event boundary* was significant, χ^2^ (1) = 13.29, *p* < .001, with a lower hit rate in the condition with event boundaries than without event boundaries. Both the main effect for *portion*, χ^2^ (1) = 14.41, *p* = .230, and the interaction of both factors, χ^2^ (1) = 0.51, *p* = .473, were not significant (see Fig. 5B).

#### Sensitivity (d’)

The interaction of *portion* and *event boundary* was significant, χ^2^ (1) = 5.81, *p* = .016, with the lowest sensitivity in the condition depicting the ends of event boundaries (Fig. 5C). In addition, the main effect for event boundary was significant, χ^2^ (1) = 9.88, *p* = .002, with lower sensitivity in the condition with event boundaries than without event boundaries. The main effect for *portion* was significant, χ^2^ (1) = 16.53, *p* < .001, with lower sensitivity in the end portion condition.

#### Response bias

The main effect of event boundary was not significant, χ^2^ (1) = 3.42, *p* = 0.064. The main effect of *portion* was significant, χ^2^ (1) = 32.32, *p* < .001, with a lower criterion for the end portions. Further, the interaction of both factors was not significant, χ^2^ (1) = 0.10, *p* = .749, was significant (see Fig. 5D). Please see Appendix B (Online Supplemental Material) for a detailed view of the estimates for each dependent variable.

### Discussion

Consistent with our hypotheses, we observed a significant interaction between *portion* and *event boundary* for false alarms, indicating that event completion occurred more for summaries including the end portions of event boundaries. Sensitivity (i.e., d’) also mirrored this effect, indicating the lowest sensitivity in the condition depicting the ends of event boundaries. The results align with the backward inferences hypothesis, suggesting that end portions of an event boundary led to more event completion. Previous studies (Kominsky et al. 2021; Papenmeier et al., 2019; Strickland & Keil, 2011) researched backwards inferences as they relate to what happens after a missing segment. In our stimuli, the endings and beginnings signify either the resolution to each micro-event (i.e., preparing tea) in the scaled-up series of events (i.e., preparing breakfast) or the start of the event, respectively. We questioned whether presenting only this information would lead to a complete representation. Therefore, the backwards inference, basically filling in the previous information, relates to what is missing “before” the consequent. In our case, the “consequent” would be the ending of the event. So, filling in effect occurs in the absence of the “antecedent.” We also relate this completion to causality as the ending of the event acts as a causal connection. We should also note that the false alarm rate in Experiment 2 is generally lower than in Experiment 1. Several explanations may account for this observation. One crucial factor is the difference in the experimental designs. In Experiment 2, we only used events that included a macro end and varied the portion (beginning vs. end) of a given event. Further, we used a different participant pool. While these differences can account for differences in the general false alarm rate, the within-subjects design controls for any potential influences on the main results of this study.

## General discussion

The event completion phenomenon has been consistently demonstrated in prior research for micro-events (i.e., kicking a ball) (Brockhoff et al., 2016; Strickland & Keil, 2011), but how it applies to macro-events (i.e., cleaning a room) has received less attention. As a macro-event can be considered a combination of multiple micro-events, it assumes a different structure. In these kinds of stimuli, multiple micro-events are linked on a more conceptual level. Considering that missing information is what drives the event completion process, using event summaries with incomplete information offers a valuable tool to study completion of macro-level events. In this study, we showed that the event completion phenomenon also holds for macro-events. In addition, although the importance of event boundaries for perceptual (Huff et al., 2012) and cognitive processes (Huff et al., 2014) as well as for the comprehension of event summaries (Schwan & Garsoffky, 2004) has been demonstrated, this is first evidence that event boundaries also affect the event completion phenomenon. In Experiment 1, more event completion emerged for summaries depicting both event boundary information and also a macro end. This is indicated with higher false alarms and lower d’ scores. At first glance, this result may be contrary to the literature, which found higher retention for event boundaries. In most event perception studies, both event boundary and non-event boundary information are present in a single stimulus (as they are presented within the natural course of the action). However, we were interested in the representations of events with missing information, and whether completion of events depends on the nature of information. We further speculated that event completion depends on whether the event has a macro end. So, the macro end (overarching end) influences the memorability of information related to event boundaries. This provides a new insight on the boundary conditions for event perception.

We should stress that event completion and event memory are different constructs. Viewers remember event boundaries better when events are presented in their full format, but event completion studies show the opposite pattern: coherent representation obtained with macro ends turns event boundary advantage around, leading to more false alarms to missing segments. In the context of event completion, stimuli leading to high false alarms are expected to lead to more coherent representations in the face of incomplete information. Therefore, higher false alarm rates and lower sensitivity (d’) observed for event boundaries suggest that those in fact have more information value, promoting a complete representation in a sparse manner. This is also consistent with previous research, which showed that event summaries including only boundary information were perceived as similar to complete events (Schwan & Garsoffky, 2004).

Contrary to our hypotheses, macro end did not yield a significant effect on detection, but an interaction emerged between event boundary and macro end. This suggested that macro end affects event completion more when attached to an event boundary summary. This is a novel finding showing first that the role of causal continuation observed for micro-level events also holds true for macro-level events. Secondly, macro-level events benefit more from an overarching ending when the event boundaries are depicted. This connects event cognition theories to the event completion phenomenon through the role of boundaries in event summaries.

How can the macro end play a role in event completion? For example, in the breakfast scenario, while leaving the kitchen with a full tray might be one of the many scenarios that could have implied a coherent continuation, it still provides a bigger signal compared to the absence of such an ending. While macro ends do not lead to event completion on their own, if an event summary containing boundary information ends with achievement of multiple micro goals, it leads to more event completion. Considering literature where completed goals remain more available than neutral goals (Lutz & Radvansky, 1997), it is logical to assume that the existence of a macro end provides a more coherent event model. Therefore, simply the end of the last micro-event does not send a signal as strong as the end of multiple micro-events.

In Experiment 2, we observed an interaction between portion and event boundary showing more event completion for summaries formed out of ends of event boundaries. We also found a main effect of endings independently of event boundaries. This shows that endings also lead to more event completion when there is a macro end. This finding is consistent with the findings of the literature, where bounded events lead ends to be prioritized (Ji & Papafragou, 2020), and disruptions in the ends are less observable than midpoints (Ji & Papafragou, 2022). We believe our findings of higher false alarm rates (more event completion) are driven also by the boundedness of the events we used. Since event boundaries were defined using segmentation data of a large population (Bezdek et al., 2023), events we used were temporally sliceable (bounded). Such manipulation also allowed event ends to lead to more completion, independently of event boundaries as well. However, we claim that this effect is more strongly driven by the endings of event boundaries, new events are only perceived at event boundaries. This may suggest that event completion is based more on backwards inferences. A backward inference is based on deduction of precedent information based on the antecedent (Papenmeier et al., 2019; Schmalhofer et al., 2002). Consequently, it is based mostly on a top-down process, where previous information is needed to infer missing information. As previous research mostly focused on backwards inferences made in written narratives (Graesser et al., 1994; Haviland & Clark, 1974; Kintsch, 1988, 1998; Singer, 1996) and picture stories (Huff et al., 2020; Magliano et al., 2016; Magliano et al., 2017), this topic is open to investigation in dynamic visual events. We should also note however that backwards inferences and predictive inferences are not mutually exclusive. They are both activated while forming event models. Our results suggest that the event completion phenomenon is rather more sensitive to backwards inferences, which lead to higher false alarms.

Our findings may appear contradictory to some previous research which found a beginning advantage for certain event categories (Teigen et al., 2017). While researchers argued for more significance and attentional demand in the case of event beginnings, this was observed for historical events, which might have a different nature compared to our stimuli. In our case, we used everyday events. As we can argue that the role of beginnings may not be generalized to all event types, everyday event routines might provide a more bounded structure where the ends also provide just as much, if not more information than the beginnings. Though we should be cautious about concluding about how event endings lead to completion for macro-events as our manipulation applies to each micro-event (i.e., dusting a shelf). We can suggest that the end portion of an event boundary for any micro-level event holds a causal nature, and the sum of these causal connections leads to the representation of a macro-level event. Therefore, a macro-event formed out of causal connections leads to more event completion.

The results of Experiment 2 apply both to EST (Radvansky & Zacks, 2011; Zacks et al., 2007) and the Event Indexing Model (Zwaan et al., 1995; Zacks et al., 2007), both of which propose causality as one of the dimensions people monitor to decide on event boundaries. We add to these theories by making the distinction that causality may be the underlying factor driving event completion via event boundaries. And this is evident from boundary ends leading to more false alarms and lower detection performance. Our results can also be tied to the *Event Horizon Model*, which suggests that people track the causal structure of events, and causality helps segment events (Radvansky, 2012; Radvansky & Zacks, 2014). Research indicates that when causal structure is absent, the perception of temporal structure also suffers. If there is a causal break in a text, reading times get longer, suggesting that the absence of a causal connection increases the cognitive load, leading to a new event model (Zwaan et al., 1995). In addition, people remember causally connected information better and faster, promoting memory retrieval (Radvansky & Copeland, 2000, 2006; Trabasso & van den Broek, 1985). Therefore, end portions of event boundaries may signify causality for each micro) event and lead to increased event completion on a macro level.

### Limitations and future directions

Although our results are consistent with the event completion literature, some characteristics of our study (regarding operationalization and stimuli) might have influenced the results. The event summaries depicted a macro end of a naturalistic action, or this ending was absent. This represents a variation of classical event completion research, in which an event either shows a *causal* or a *non-causal continuation*. Therefore, in the classical paradigm, a non-causal continuation condition would be required as a control. However, in our design, we manipulated the *presence* or *absence* of an ending. So, in our case, the complete video with the macro end can be considered as the control condition. Further, the event boundary information length was chosen to be 8 s. Future research should test the limits of this process to indicate the optimal time needed. Another limitation can be linked to the absence of sound in our videos. Research showed that audio also uses the same working memory capacity and may lead to phenomena such as edit blindness (Meitz et al., 2020). Future research should investigate the role of diegetic sound in event completion.

In our stimuli, most coarse boundaries also involved ends of fine boundaries with them. For example, in one of our videos, the actor folded multiple clothes (fine events: folding t-shirt, pants etc., coarse event: folding clothes) before folding towels (fine events: folding the first towel, folding the second towel etc., coarse event: folding towels). As can be observed from the examples, each coarse event can end with a fine event, but the ending of a fine event (folding last piece of clothing) can coincide with a coarse event only if a new category of events (folding towels) begins. We take such structures as evidence for enclosure in event structures. Enclosure is a tool to measure hierarchical structure between coarse and fine event boundaries (Hard et al., 2011; Sargent et al., 2013). Granted there is a hierarchical structure between grain sizes, coarse event boundaries would enclose fine events, oftentimes occurring around similar points. Even though we haven’t measured the enclosure between grains, the ending of coarse events still marks beginnings of a new coarse event, despite coinciding with ending of a fine event. However, more research is needed for the manipulation of complex event structure, and future studies can compare event completion with low and high enclosures in event structure.

Our study has implications for macro-events, for which the order is not strictly defined. While micro-events are order-dependent (breaking an egg must come before making an omelet), micro-events can be reorganized in alternative ways (making a coffee vs. an omelet). Therefore, it is important to distinguish the conditions for which event completion might apply. It is an open question whether alternative orders for the same action can lead to event completion in similar ways. Future studies can manipulate the order of the same action between conditions to understand event completion in macro-events better.

Event completion in general can also be understood in terms of a top-down effect of event schema. Event schema holds the semantic information of commonalities across multiple events in terms of event classes and structures of information storing (Radvansky & Zacks, 2011). Event schema drives the expectations by guiding event models in terms of causality, characters, location, time, goals or intentions. Results of our studies are not enough to claim that more event completion occurs purely due to causal inferences. In fact, it could well be the case that inferences towards causality are built upon tracking other situational dimensions. However, it is logical to assume that causality and remaining situational dimensions are tracked together to make inferences about missing information, in terms of event completion. Future studies could manipulate multiple situational dimensions to understand event completion better.

### Practical implications

Event completion, by its nature, is a form of false memory. It is a distortion of memory that affects the encoding of parts that are seen from parts that were missing in the presentation of a stimulus. Being aware of this process especially becomes important with modern media, as event summaries, or videos created with parts of action are used to represent the entirety of action (trailers, game summaries of NBA or football games, etc.) (Huff et al., 2017). Even though they allow for a time-saving summary of larger events, it must be taken account that memory towards unseen parts might be misleading the information that is successfully encoded with the video. Further research can study how viewers “hallucinate” based on event summaries as they would lead to certain expectations. Research can also inquire whether these types of false memory pertain to micro or macro-level events. We inform viewers by suggesting that endings of events lead to this process and, pre-segmented video summaries, or seeing only the endings of each event might mislead the event schema of what happened. This could be observed with lecture summaries that benefit from edits with pre-segmented parts, that highlight the important parts of lecture.

## Conclusions and outlook

We present the first evidence that event completion occurs for macro-level events consisting of smaller events, through the presence of a macro end. We also link event completion to the perception of event structure since event boundaries include end and beginning of action. Lastly, we suggest that event endings provide critical information for event completion than event beginnings and pinpoint the source of event completion within the event structure.

## Supplementary Information

Below is the link to the electronic supplementary material.

Supplementary file1 (DOCX 25 KB)

## Data Availability

The stimuli, data, pre-registration files, codes and analyses for the first and second experiments can be found in the OSF link: https://osf.io/r28zd/

## Code availability

Experiment files of PsychoPy are also available in OSF.
