# Supplementary material for: How does it end? Endpoints of boundaries lead to completion in macro-events
Source: Mem Cognit. 2024 Nov 15;53(5):1380–95. doi: 10.3758/s13421-024-01657-x (PMC12307519; doi:10.3758/s13421-024-01657-x)
Supplement: Supplementary file 1 — Supplementary file1 (DOCX 25 KB) [file 13421_2024_1657_MOESM1_ESM.docx]

APPENDIX A

Estimates of the models fitted for the results of Experiment 1 (false alarm rate, hit rate, detection performance, and response bias).

|  | **False alarm rate** | |
| --- | --- | --- |
| *Predictors* | *Estimates* | *p* |
| (Intercept) | 0.32  (0.28 – 0.37) | <0.001 |
| Event boundary: Without | -0.04  (-0.07 – -0.01) | 0.008 |
| Macro end: Absent | -0.05  (-0.11 – 0.01) | 0.108 |
| Event boundaryWithout:Macro endAbsent | 0.05  (0.00 – 0.09) | 0.039 |
| **Random Effects** | | |
| σ^2^ | 0.01 | |
| τ_00_ _id_ | 0.03 | |
| ICC | 0.75 | |
| N _id_ | 137 | |
| Observations | 274 | |
| Marginal R^2^ / Conditional R^2^ | 0.012 / 0.757 | |

|  | **Hit rate** | |
| --- | --- | --- |
| *Predictors* | *Estimates* | *p* |
| (Intercept) | 0.76  (0.73 – 0.79) | <0.001 |
| Event boundary: Without | 0.04  (0.01 – 0.06) | 0.002 |
| Macro end: Absent | 0.01  (-0.03 – 0.06) | 0.515 |
| Event boundaryWithout:Macro endAbsent | -0.03  (-0.07 – 0.00) | 0.069 |
| **Random Effects** | | |
| σ^2^ | 0.01 | |
| τ_00_ _id_ | 0.01 | |
| ICC | 0.67 | |
| N _id_ | 137 | |
| Observations | 274 | |
| Marginal R^2^ / Conditional R^2^ | 0.011 / 0.674 | |

|  | **Detection performance (d’)** | |
| --- | --- | --- |
| *Predictors* | *Estimates* | *p* |
| (Intercept) | 0.32  (0.28 – 0.37) | <0.001 |
| Event boundary: Without | -0.04  (-0.07 – -0.01) | 0.008 |
| Macro end: Absent | -0.05  (-0.11 – 0.01) | 0.108 |
| Event boundaryWithout:Macro endAbsent | 0.05  (0.00 – 0.09) | 0.039 |
| **Random Effects** | | |
| σ^2^ | 0.01 | |
| τ_00_ _id_ | 0.03 | |
| ICC | 0.75 | |
| N _id_ | 137 | |
| Observations | 274 | |
| Marginal R^2^ / Conditional R^2^ | 0.012 / 0.757 | |

|  | **Response bias (c)** | |
| --- | --- | --- |
| *Predictors* | *Estimates* | *p* |
| (Intercept) | -0.12  (-0.20 – -0.05) | 0.002 |
| Event boundary: Without | 0.02  (-0.05 – 0.09) | 0.579 |
| Macro end: Absent | 0.02  (-0.10 – 0.14) | 0.731 |
| Event boundaryWithout:Macro endAbsent | 0.00  (-0.10 – 0.11) | 0.955 |
| **Random Effects** | | |
| σ^2^ | 0.05 | |
| τ_00_ _id_ | 0.07 | |
| ICC | 0.56 | |
| N _id_ | 137 | |
| Observations | 274 | |
| Marginal R^2^ / Conditional R^2^ | 0.002 / 0.565 | |

APPENDIX B

Estimates of the models fitted for the results of Experiment 2 (false alarm rate, hit rate, detection performance, and response bias).

|  | **False alarm rate** | |
| --- | --- | --- |
| *Predictors* | *Estimates* | *p* |
| (Intercept) | 0.17  (0.14 – 0.20) | <0.001 |
| Event boundary: Without | 0.01  (-0.02 – 0.04) | 0.556 |
| Portion: End | 0.08  (0.06 – 0.11) | <0.001 |
| Event boundaryWithout:PortionEnd | -0.05  (-0.09 – -0.01) | 0.028 |
| **Random Effects** | | |
| σ^2^ | 0.01 | |
| τ_00_ _id_ | 0.01 | |
| ICC | 0.51 | |
| N _id_ | 90 | |
| Observations | 360 | |
| Marginal R^2^ / Conditional R^2^ | 0.055 / 0.541 | |

|  | **Hit rate** | |
| --- | --- | --- |
| *Predictors* | *Estimates* | *p* |
| (Intercept) | 0.75  (0.72 – 0.78) | <0.001 |
| Event boundary: Without | 0.03  (0.00 – 0.06) | 0.039 |
| Portion: End | 0.01  (-0.03 – 0.04) | 0.733 |
| Event boundaryWithout:PortionEnd | 0.02  (-0.03 – 0.06) | 0.474 |
| **Random Effects** | | |
| σ^2^ | 0.01 | |
| τ_00_ _id_ | 0.01 | |
| ICC | 0.49 | |
| N _id_ | 90 | |
| Observations | 360 | |
| Marginal R^2^ / Conditional R^2^ | 0.021 / 0.502 | |

|  | **Detection performance (d’)** | |
| --- | --- | --- |
| *Predictors* | *Estimates* | *p* |
| (Intercept) | 1.89  (1.73 – 2.06) | <0.001 |
| Event boundary: Without | 0.04  (-0.12 – 0.20) | 0.605 |
| Portion: End | -0.37  (-0.53 – -0.21) | <0.001 |
| Event boundaryWithout:PortionEnd | 0.28  (0.05 – 0.51) | 0.016 |
| **Random Effects** | | |
| σ^2^ | 0.30 | |
| τ_00_ _id_ | 0.34 | |
| ICC | 0.53 | |
| N _id_ | 90 | |
| Observations | 360 | |
| Marginal R^2^ / Conditional R^2^ | 0.040 / 0.553 | |

|  | **Response bias (c)** | |
| --- | --- | --- |
| *Predictors* | *Estimates* | *p* |
| (Intercept) | 0.15  (0.08 – 0.23) | <0.001 |
| Event boundary: Without | -0.06  (-0.14 – 0.02) | 0.126 |
| Portion: End | -0.17  (-0.24 – -0.09) | <0.001 |
| Event boundaryWithout:PortionEnd | 0.02  (-0.09 – 0.13) | 0.749 |
| **Random Effects** | | |
| σ^2^ | 0.07 | |
| τ_00_ _id_ | 0.05 | |
| ICC | 0.40 | |
| N _id_ | 90 | |
| Observations | 360 | |
| Marginal R^2^ / Conditional R^2^ | 0.057 / 0.434 | |
